# Up-Converting Luminescence and Temperature Sensing of Er^3+^/Tm^3+^/Yb^3+^ Co-Doped NaYF_4_ Phosphors Operating in Visible and the First Biological Window Range

**DOI:** 10.3390/nano11102660

**Published:** 2021-10-10

**Authors:** Jingyun Li, Yuxiao Wang, Xueru Zhang, Liang Li, Haoyue Hao

**Affiliations:** 1 School of Physics and Optoelectronic Engineering, Shandong University of Technology, Zibo 255000, China; li.jingyun@outlook.com; 2 Department of Physics, Harbin Institute of Technology, Harbin 150001, China; wangyx@hit.edu.cn (Y.W.); xrzhang@hit.edu.cn (X.Z.)

**Keywords:** luminescent materials, rare earth doped materials, optical thermometry, luminescence intensity ratio

## Abstract

Accurate and reliable non-contact temperature sensors are imperative for industrial production and scientific research. Here, Er^3+^/Tm^3+^/Yb^3+^ co-doped NaYF_4_ phosphors were studied as an optical thermometry material. The typical hydrothermal method was used to synthesize hexagonal Er^3+^/Tm^3+^/Yb^3+^ co-doped NaYF_4_ phosphors and the morphology was approximately rod-like. The up-conversion emissions of the samples were located at 475, 520, 550, 650, 692 and 800 nm. Thermo-responsive emissions from the samples were monitored to evaluate the relative sensing sensitivity. The thermal coupled energy level- and non-thermal coupled energy level-based luminescence intensity ratio thermometry of the sample demonstrated that these two methods can be used to test temperature. Two green emissions (520 and 550 nm), radiated from ^2^H_11/2_/^4^S_3/2_ levels, were monitored, and the maximum relative sensing sensitivities reached to 0.013 K^−1^ at 297 K. The emissions located in the first biological window (650, 692 and 800 nm) were monitored and the maximum relative sensing sensitivities reached to 0.027 (*R*_692/650_) and 0.028 K^−1^ (*R*_692/800_) at 297 K, respectively. These results indicate that Er^3+^/Tm^3+^/Yb^3+^ co-doped NaYF_4_ phosphors have potential applications for temperature determination in the visible and the first biological window ranges.

## 1. Introduction

Temperature (*T*) is an important physical parameter in many fields, like scientific research, industrial production and biotherapy. Accurate *T* can usually be detected via contacting the temperature sensors, such as with thermal resistance, thermocouples and semiconductor temperature sensors. However, these temperature sensors limit their applications in temperature exploration when the measured objects are displayed in electromagnetic noise environments or beings. Thus, it is crucial to explore non-contact temperature sensors, such as IR thermography, Raman spectroscopy, and luminescence [1,2,3,4,5,6]. The non-contact temperature sensor based on temperature-dependent luminescence properties has drawn a lot of attention for its high resolution, stability and repeatability [7].

For lanthanide ion-doped materials, their luminescence intensity, peak position, emission band width, emission lifetime and luminescence intensity ratio (LIR) have been extensive researched for non-contact optical thermometry [7,8,9,10,11]. One of the most interesting developments is LIR-based temperature sensing as it is not influenced by pressure, light source and/or atmosphere [12]. Er^3+^ doped nanomaterials are promising in LIR-based temperature sensing for their evident green emissions from ^2^H_11/2_/^4^S_3/2_ and excellent thermal coupling properties [13,14,15,16]. Thus, we choose Er^3+^ as one of the doped rare earth ions and the green emissions can be used as the detected signal in the visible range for thermometry. However, the green emissions have obvious absorption and limited penetration depth in biological tissues [17,18]. Therefore, the selected emissions shall be located in biological windows when the object is located in biological tissues [18]. Under 980 nm laser excitation, Tm^3+^ can emit red (650 nm) and near-infrared emissions (692 and 800 nm) [19], which can be used as the detected signal in the first biological window (650–1000 nm).

The non-thermal coupled levels have also been used in LIR thermometry because of their high sensing sensitivity [20,21,22]. For example, high relative sensing sensitivity (0.0034 K^−1^) was obtained in NaLuF_4_:Yb/Er/Ho nano-rods at 503 K, which is based on the emissions at 659 and 547 nm [23]. Therefore, non-thermal coupled level-based LIR thermometry is an excellent method for temperature measuring, which can promote relative sensing sensitivity and select suitable wavebands.

Herein, Er^3+^/Tm^3+^/Yb^3+^ were selected as the doped ions, with which Er^3+^/Tm^3+^ acted as the emitting centers and Yb^3+^ acted as the sensitizer. In this study, we selected hexagonal phase NaYF_4_ as the host matrix due to its relatively excellent chemical and thermal stabilities and its low phonon energy (~370 cm^−1^) [24]. The rod-like NaYF_4_: Er^3+^, Tm^3+^, and Yb^3+^ phosphors were prepared through the hydrothermal method. The emissions (450–850 nm) from Er^3+^/Tm^3+^/Yb^3+^ co-doped NaYF_4_ phosphors were systemically investigated. High relative temperature sensitivity was achieved via choosing suitable LIR of green emissions and the emissions located in the first biological window. We can take advantages of this multi-band noninvasive thermometry in harsh environments or biological tissues.

## 2. Experimental Details

### 2.1. Sample Preparation

#### 2.1.1. Materials

Y(NO_3_)_3_·6H_2_O (99.9%), Yb(NO_3_)_3_·6H_2_O (99.9%), Tm(NO_3_)_3_·6H_2_O (99.9%), Er(NO_3_)_3_·6H_2_O (99.9%) were all purchased from the Jining Zhong Kai New Type Material Science Co., Ltd, Jining, China. Ammonium fluoride (AR), sodium hydroxide (AR), oleic acid (AR) and cyclohexane (AR) were purchased from the Tianjin Tianli Chemical reagent Co. Ltd, Tianjin, China. All the chemicals were used directly without further purification.

#### 2.1.2. Preparation of NaYF_4_: 2 mol% Er^3+^, 20 mol% Yb^3+^ and NaYF_4_: 2 mol% Er^3+^, 0.5 mol% Tm^3+^, 20 mol% Yb^3+^ Phosphors

The Er/Yb co-doped NaYF_4_ phosphors were prepared using the hydrothermal method. The preparation processes are described below. First, calculated amounts of sodium hydroxide were dissolved into 2 mL deionized water. Second, 10 mL absolute ethyl alcohol and 18 mL oleic acid were added to the nitrate solution and then stirred for 5 min at room temperature to form a faint yellow solution. Third, 5 mL aqueous solution which contained calculated amount of Y(NO_3_)_3_·6H_2_O, Yb(NO_3_)_3_·6H_2_O and Er(NO_3_)_3_·6H_2_O was added. Then 5 mL ammonium fluoride aqueous was immediately added. After stirring for 30 min at room temperature, the mixed solution was transferred into a 50 mL autoclave and heated at 180 °C for 12 h in a vacuum drying oven. After cooling down to room temperature and adding a certain percentage of ethanol and cyclohexane, the khaki suspension was centrifuged (8000 rpm, 2 min) for collection and washed three times with ethanol and deionized water. Finally, the phosphors were obtained after drying at 60 °C for 10 h. The Er/Tm/Yb co-doped NaYF_4_ phosphors were prepared using the same method, except for the amount of Y(NO_3_)_3_·6H_2_O, Yb(NO_3_)_3_·6H_2_O, Tm(NO_3_)_3_·6H_2_O and Er(NO_3_)_3_·6H_2_O.

### 2.2. Instruments

X-ray diffraction (XRD) patterns of the sample were tested using an X-ray diffractometer (D8–02, BrukerAXS, Karlsruhe, Germany). The morphology was tested using a transmission electron microscope (TEM: Tecnai G2 F20, FEI, Hillsboro, OR, USA). The spectra of the samples were tested through the iHR550 grating spectrograph (iHR550, Horiba, Paris, France). The 980 nm laser used to excite the sample was purchased from the Beijing Kipling Photoelectric technology Co., Ltd, Beijing, China. (model: K980F14CC-10.00 W). For the thermometry experiments, we introduced a Linkam THMS 600 heating stage to heat the sample. Then the temperature of the sample was measured by thermocouple. The spectra of the sample at certain temperatures were acquired using the iHR550 grating spectrometer (iHR550, Horiba, Paris, France).

## 3. Results and Discussions

### 3.1. XRD Analysis

**Figure 1:**
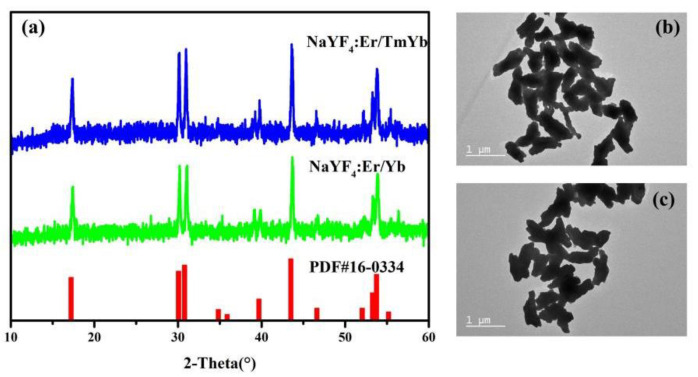
(**a**) XRD patterns of NaYF_4_: Er/Yb and NaYF_4_: Er/Tm/Yb phosphors; TEM images of (**b**) NaYF_4_: Er/Yb and (**c**) NaYF_4_: Er/Tm/Yb phosphors (the scale bare is 1 μm).

The XRD patterns of NaYF_4_: Er/Yb and NaYF_4_: Er/Tm/Yb phosphors are presented in Figure 1a. The XRD patterns of the samples can be indexed to hexagonal NaYF_4_ crystal (the JCPDS standard card no. 16-0334), indicating that the dopants (Er, Tm and Yb ions) are successfully incorporated into the host lattice and do not cause significant changes to the crystal structure. Figure 1b,c show the TEM images of the samples. Two samples’ morphologies are approximately rod-like. The lengths of the rods are ~890 nm and the length–diameter ratios are ~3.3.

### 3.2. Power Dependent Up-Conversion Luminescence

**Figure 2:**
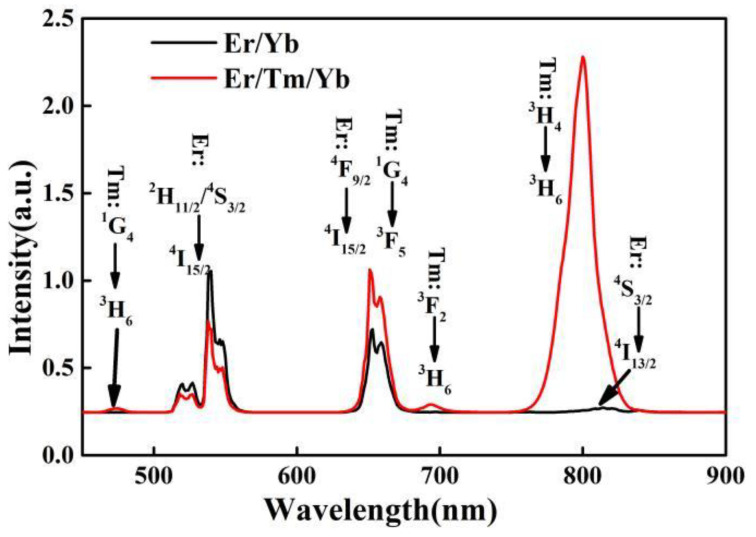
Up-conversion spectra of the samples under 980 nm laser excitation (5 W/cm^2^).

**Figure 3:**
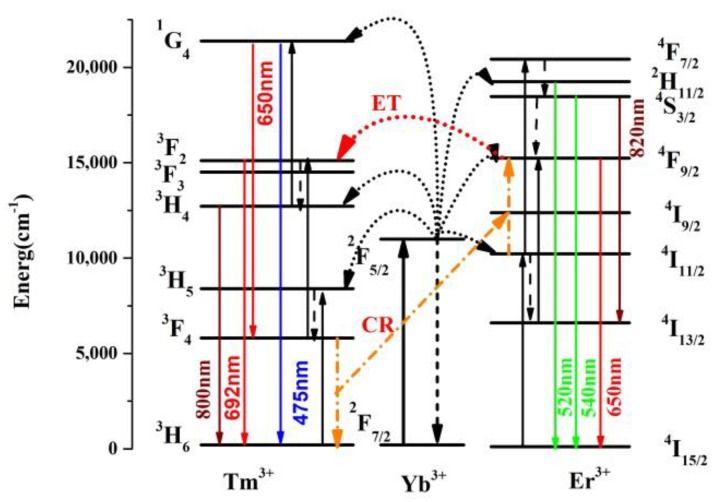
Schematic energy levels and the possible up-conversion processes in Er^3+^, Tm^3+^, Yb^3+^ co-doped materials.

To investigate the emission spectra of the synthesized Er/Yb and Er/Tm/Yb co-doped NaYF_4_ phosphors, the samples were excited under a 980 nm semiconductor laser. As shown in Figure 2, the emissions radiated from Er/Yb co-doped NaYF_4_ phosphors are located at 520, 550, 650 and 820 nm, which originated from the transitions of ^2^H_11/2_ → ^4^I_15/2_ (Er^3+^: 525 nm), ^4^S_3/2_ → ^4^I_15/2_ (Er^3+^: 550 nm), ^4^F_9/2_ → ^4^I_15/2_(Er^3+^: 650 nm) and ^4^S_3/2_ → ^4^I_13/2_ (Er^3+^: 820 nm), respectively. The emissions radiated from Er/Tm/Yb co-doped NaYF_4_ phosphors are located at 475, 520, 550, 650, 692 and 800 nm. The extra emissions are derived from the transitions of ^1^G_4_ → ^3^H_6_ (Tm^3+^: 475 nm), ^1^G_4_ → ^3^F_4_ (Tm^3+^: 650 nm), ^3^F_2_ → ^3^H_6_ (Tm^3+^: 692 nm) and ^3^H_4_ → ^3^H_6_ (Tm^3+^: 800 nm). It can be found from the emission spectrum of Er/Tm/Yb co-doped NaYF_4_ phosphors that the 820 nm emission is almost invisible because of the intense 800 nm emission peak. The detailed energy levels and the possible up-conversion processes in Er^3+^, Tm^3+^, Yb^3+^ co-doped materials are displayed in Figure 3. Under the excitation of a 980 nm laser, the energy level Yb^3+^: ^2^F_5/2_ is populated through ground state absorption (GSA). The energy level Er^3+^: ^4^I_13/2_ is populated through the energy transferred from Yb^3+^ (^4^I_15/2_ (Er^3+^) +^2^F_5/2_ (Yb^3+^) → ^4^I_11/2_ (Er^3+^) +^2^F_7/2_ (Yb^3+^)). The energy level Er^3+^: ^4^F_7/2_ is populated via the energy transferred from Yb^3+^ (^4^I_11/2_ (Er^3+^) +^2^F_5/2_ (Yb^3+^) → ^4^F_7/2_ (Er^3+^) +^2^F_7/2_ (Yb^3+^)). After non-radiative relaxation from energy level ^4^F_7/2_ to ^2^H_11/2_, ^4^S_3/2_, and further to ^4^F_9/2_, green (^2^H_11/2_,^4^S_3/2_ → ^4^I_15/2_) red (^4^F_9/2_ → ^4^I_15/2_) and the emissions located at 820 nm (^4^S_3/2_ → ^4^I_1__3__/2_) occur. In addition, the population of ^4^F_9/2_ state can be obtained via another process: ^4^I_13/2_(Er^3+^) +^2^F_5/2_ (Yb^3+^) → ^4^F_9/2_ (Er^3+^) +^2^F_7/2_ (Yb^3+^), where the energy level Er^3+^: ^4^I_13/2_ is accumulated from Er^3+^: ^4^I_11/2_ through non-radiative relaxation. The energy level Tm^3+^: ^3^H_5_ is populated through the energy transferred from Yb^3+^ (^3^H_6_ (Tm^3+^) +^2^F_5/2_ (Yb^3+^) → ^3^H_5_ (Tm^3+^) +^2^F_7/2_ (Yb^3+^)). The energy levels Tm^3+^: ^3^F_2_ and ^1^G_4_ are populated via the energy transferred from Yb^3+^ (^3^F_4_ (Tm^3+^) +^2^F_5/2_ (Yb^3+^) → ^3^F_2_ (Tm^3+^) +^2^F_7/2_ (Yb^3+^) and ^3^H_4_ (Tm^3+^) +^2^F_5/2_ (Yb^3+^) → ^1^G_4_ (Tm^3+^) +^2^F_7/2_ (Yb^3+^)). Blue and red, the emissions located at 692 and 800 nm, are radiated from the transitions ^1^G_4_ → ^3^H_6_, ^1^G_4_ → ^3^F_4_, ^3^F_2_ → ^3^H_6_ and ^3^H_4_ → ^3^H_6_, respectively. Apart from the up-conversion processes between Er-Yb and Tm-Yb, the energy transfer (ET: ^4^F_9/2_ (Er^3+^) → ^3^F_2_(Tm^3+^)) and cross relaxation (CR: ^3^F_4_ (Tm^3+^) + ^4^I_11/2_ (Er^3+^) → ^3^H_6_ (Tm^3+^) + ^4^F_9/2_ (Er^3+^)) occur. The ET and CR can increase the emission intensities at 692 and 650 nm, respectively. The decrease of the green emission is caused by CR, which can reduce the energy excited to ^4^F_7/2_ (Er^3+^).

**Figure 4:**
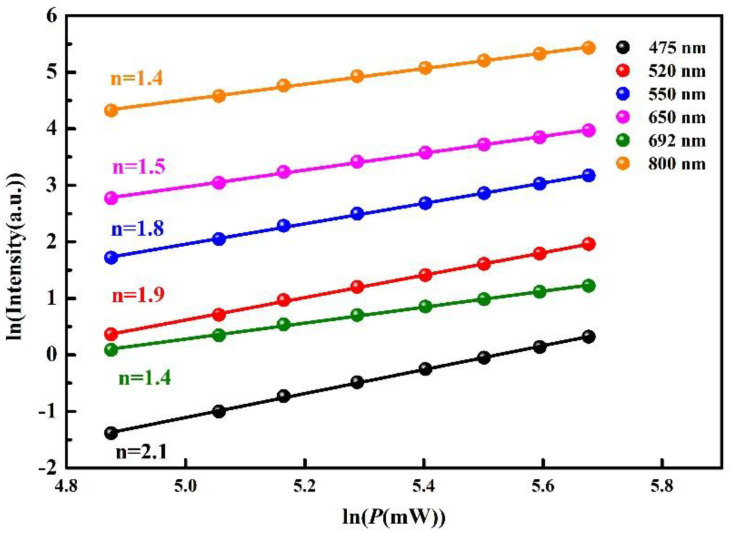
Ln-ln plot of emission intensity against the excitation power for NaYF_4_:Er/Tm/Yb phosphors.

In order to obtain the number of photons required for up-conversion processes, the variation law (*I*∝*P^n^*) of excitation power (*p*) dependent emission intensity (*I*) is shown in Figure 4. The slopes of the fitting line in the ln*I*-ln*P* plot represents the photons (*n*) participating in the up-conversion processes [25]. The values of *n* are 2.1, 1.9, 1.8, 1.5, 1.8, 1.5, 1.4 and 1.4 for 475, 520, 550, 650, 692 and 800 nm emissions, respectively. Therefore, the emission of blue comes from the three-photon process and the other emissions derive from the two-photon process. The participating photons, calculated from the slopes, are consistent with the up-conversion processes in Figure 3.

### 3.3. Temperature-Dependent Up-Conversion Luminescence

**Figure 5:**
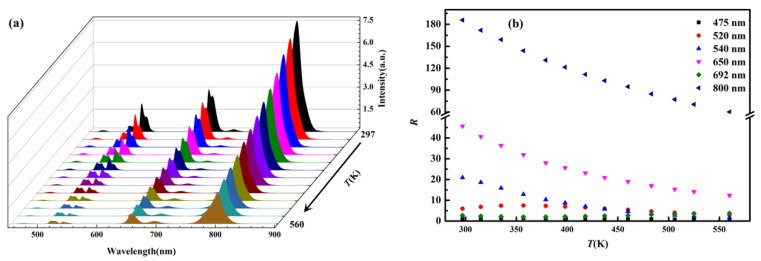
(**a**) Up-conversion luminescence of the sample at different temperatures; (**b**) temperature-dependent integrated intensity of the emissions located at different wavelengths.

In order to analyze the variation law of up-conversion luminescence as the temperature changes, the emission spectra were obtained when the NaYF_4_: Er^3+^, Tm^3+^, Yb^3+^ phosphors were heated by heating stage. The up-conversion spectra of the sample at different temperatures are displayed in Figure 5a and the temperature-dependent integrated intensity of the emissions located at different wavelengths are displayed in Figure 5b. As can be seen, most of the emissions radiated from the sample decrease with increasing temperature except for the emissions located at 520 and 692 nm. The reason for emission decreases is that the non-radiative transition increases with the increase of temperature. However, the emission increases at 520 and 692 nm are due to the thermal excitation from the adjacent lower energy levels (^4^S_3/2_ → ^2^H_11/2_ (Er^3+^)/^3^H_4_ → ^3^F_2_ (Tm^3+^)).

**Figure 6:**
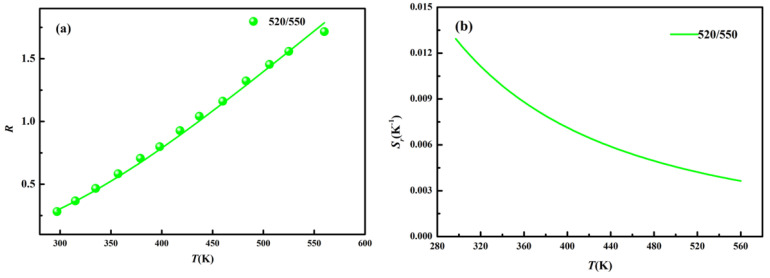
(**a**) Temperature dependent *R*_520/550_; (**b**) *S*_r_ of *R*_520/550_.

To measure temperature via LIR, we chose the emissions located at 520 and 550 nm as the detected signals in the visible range and the emissions located at 650, 692 and 800 nm as the signals in the first biological window. For thermal coupled energy levels, the relationship between luminescence intensity ratio (520 and 550 nm) and temperature can be mathematically expressed as follows [26,27]

(1) $$LIR=\frac{I_{high}}{I_{low}}=Cexp\left(-\frac{\Delta E}{kT}\right)$$

where *I_high_* and *I_low_* are the integrated intensities of the green emissions corresponding to the transition of high energy level to ground state (^2^H_11/2_ → ^4^I_15/2_) and low energy level to ground state (^4^S_3/2_ → ^4^I_15/2_). ΔE is the energy gap between high and low energy levels. *k* is the Boltzmann constant. *C* is a parameter related to the degeneracy, the radiative probabilities of the transitions and the angular frequency [26,27]. Using the integrated areas under the 520 and 550 nm bands and applying Equation (1), a perfect fit (R > 0.99) to the determined band intensity ratio was obtained. The temperature-dependent LIR of 520 and 550 nm (*R*_520/550_) is shown in Figure 6a, in which the fitting function is *R* = 13.9exp(−1141/*T*). In order to compare the thermometry ability with other research, we calculated the relative sensing sensitivity (*S*_r_) using the expression that follows [28]

(2) $$S_{r}\;=\;\frac{1}{R}\;\frac{dR}{dT}$$

The temperature dependent *S*_r_ of *R*_520/550_ is shown in Figure 6b and the value of relative sensing sensitivity decreases as the temperature increases from 297 to 560 K. The maximum value reaches to 0.013 K^−1^ (297 K).

**Figure 7:**
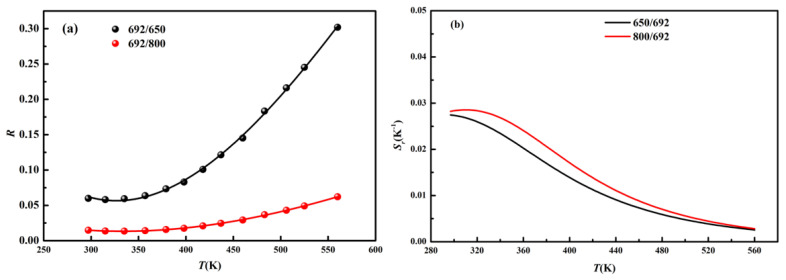
(**a**) Temperature-dependent ratios of *R*_692/650_ and *R*_692/800_; (**b**) *S*_r_ of *R*_692/650_ and *R*_692/800_.

In order to explore its thermometry ability in biological tissues, we studied the LIR of the emissions located in the first biological window (650, 692 and 800 nm). The temperature-dependent ratios of *R*_692/650_ and *R*_692/800_ are shown in Figure 7a. For the non-thermal coupled energy levels, the temperature-dependent ratios are fitted via the cubic function $R=B_{0}+B_{1}T+B_{2}T^{2}+B_{3}T^{3}$ [29] and the fitting parameters are displayed in Table 1. To evaluate the sensing capacity, the relative sensing sensitivities are calculated through Expression (2) and the sensitivity curves are displayed in Figure 7b. The relative sensing sensitivities of *R*_692/650_ and *R*_692/800_ decrease as the temperature increases from 297 to 560 K. The maximum values reach to 0.027 and 0.028 K^−1^ (297 K), respectively. To compare the thermometry capacity of NaYF_4_: Er/Tm/Yb phosphors, the relevant parameters from other research are listed in Table 2. As can be seen, the sensing sensitivities of our samples are relatively high among these works based on thermal coupled levels and non-thermal coupled levels.

## 4. Conclusions

In summary, NaYF_4_: Er^3+^, Tm^3+^, Yb^3+^ phosphors were prepared through the typical hydrothermal method. The up-conversion luminescence and temperature-dependent emissions were studied under 980 nm laser excitation. The slopes in the ln*I*-ln*P* plot are 2.1, 1.9, 1.8, 1.5, 1.8, 1.5, 1.4 and 1.4 for 475, 520, 550, 650, 692 and 800 nm emissions, respectively. This implies that the 475, 520, 550, 650, 692 and 800 nm emissions are three-photon, two-photon, two-photon, two-photon, two-photon and two-photon processes, respectively. Moreover, the thermal coupled energy level- and non-thermal coupled energy level-based LIR thermometry of the sample demonstrates that these two methods can be used to test temperature. The maximum relative sensing sensitivities of *R*_520/550_, *R*_692/650_ and *R*_692/800_ reach to 0.013, 0.027 and 0.028 K^−1^ at 297 K, respectively. The results reveal that Er/Tm/Yb co-doped NaYF_4_ phosphors have great potential in LIR-based temperature sensing at room temperature. Meanwhile, the emissions for thermometry can alter from the visible range to the first biological window based on the actual requirements.

## Figures and Tables

**Table 1:** The fitting parameters of *R*_692/650_ and *R*_692/800_.

Parameter	*R*_692/650_	*R*_692/800_
B_0_	0.94	0.17
B_1_	−0.0061	−0.0010
B_2_	1.3 × 10^−5^	1.9 × 10^−6^
B_3_	−6.7 × 10^−9^	−7.9 × 10^−10^

**Table 2:** Comparison of relative sensing sensitivities in LIR thermometry.

Materials	Wavelengths (nm)	Temperature Range (K)	*S*_r_(K^−1^) (Temperature, K)	Ref.
NaGdF_4_: Yb/Er	520/550	77–500	0.0112 (298)	[30]
La(IO_3_)_3_: Yb/Er	520/550	298–343	0.012 (298)	[31]
Ba_3_La(PO_4_)_3_: Yb/Er	520/550	298–498	0.0117 (298)	[32]
Ba_3_La(PO_4_)_3_: Yb/Tm	690/792	303–503	0.0211 (303)	[32]
YF_3_: Yb/Tm	940/800	303–345	0.008 (310)	[33]
NaErF_4_@NaGdF_4_	806/654	303–593	0.0058 (303)	[34]
GeO_2_-PbO-PbF_2_: Yb/Er	520/650	300–466	0.01 (300)	[35]
NaYbF_4_/NaYF_4_: Tm/Yb/NaYF_4_	800/1800	303–423	0.0033 (300)	[36]
Y_2_O_3_: Yb/Tm	684/490	303–573	0.0151 (445)	[37]
NaY_2_F_7_: Yb/Tm	678/700	307–567	0.016 (415)	[38]
NaYF_4_: Ho	961/1183	113–473	0.008 (367)	[39]
Na_3_ZrF_7_: Yb/Er/Tm	800/673	313–393	0.0176 (313)	[40]
NaYF_4_: Er/Tm/Yb	520/550	297–560	0.013 (297)	This work
	692/650	297–560	0.027 (297)	This work
	692/800	297–560	0.028 (297)	This work

## Data Availability

The data are available from the corresponding author upon reasonable request.
